# Epidemiology of diabetic retinopathy and maculopathy in Africa: a systematic review

**DOI:** 10.1111/j.1464-5491.2012.03756.x

**Published:** 2013-04

**Authors:** P I Burgess, I J C MacCormick, S P Harding, A Bastawrous, N A V Beare, P Garner

**Affiliations:** 1 Malawi–Liverpool–Wellcome Trust Clinical Research Programme, Queen Elizabeth Central Hospital, College of MedicineBlantyre, Malawi; 2 Department of Eye and Vision Science, University of LiverpoolLiverpool; 3 International Centre for Eye Health, Clinical Research Department, London School of Hygiene and Tropical MedicineLondon; 4 St Pauls Eye Unit, Royal Liverpool University Teaching HospitalLiverpool, UK; 5 International Health Group, Liverpool School of Tropical MedicineLiverpool, UK

## Abstract

**Abstract:**

**Aim:**

To summarize findings from studies reporting the prevalence and incidence of diabetic retinopathy and diabetic maculopathy in African countries in light of the rising prevalence of diabetes mellitus.

**Methods:**

Using a predefined search strategy, we systematically searched MEDLINE, EMBASE, Science Citation index and Conference Proceedings Citation index, African Index Medicus and the grey literature database ‘OpenSIGLE’ for studies published between January 1990 and February 2011. Included studies reported prevalence or incidence of diabetic retinopathy or diabetic maculopathy of subjects with diabetes resident in African countries.

**Results:**

Sixty-two studies from 21 countries were included: three population-based surveys; two cohort studies; five case–control studies; 32 diabetes clinic-based, nine eye clinic-based and 11 other hospital-based surveys. Included studies varied considerably in terms of patient selection, method of assessing the eye and retinopathy classification. In population-based studies, the reported prevalence range in patients with diabetes for diabetic retinopathy was 30.2 to 31.6%, proliferative diabetic retinopathy 0.9 to 1.3%, and any maculopathy 1.2 to 4.5%. In diabetes clinic-based surveys, the reported prevalence range for diabetic retinopathy was 7.0 to 62.4%, proliferative diabetic retinopathy 0 to 6.9%, and any maculopathy 1.2 to 31.1%. No obvious association between prevalence and income level of the country was detected.

**Conclusions:**

Large, community-based cross-sectional and cohort studies are needed to investigate rates and determinants of prevalence of diabetic retinopathy, incidence and progression in Africa. Consensus is needed on the most appropriate methods of identification and classification of retinopathy for research and clinical practice. Estimates of prevalence of diabetic retinopathy, proliferative diabetic retinopathy and maculopathy are comparable with recent European and American studies.

## Introduction

The International Diabetes Federation (IDF) has estimated that the number of adults with diabetes in Africa will expand by 98%, from 12.1 million in 2010 to 23·9 million in 2030 [1]—a consequence of urbanization, sedentary lifestyles, obesity, and population growth and ageing (in part as a result of successes in combating communicable diseases) [2]. Thirty-one of the 48 least economically developed countries, as defined by the United Nations, are situated in Africa [3]. The epidemic rise in diabetes therefore poses significant public health and socio-economic challenges for the continent.

Diabetes causes visual impairment through cataract and diabetic retinopathy, a progressive disease of the retinal microvasculature. Diabetic retinopathy can be broadly divided into two clinical categories: non-proliferative and proliferative diabetic retinopathy. The pathophysiology of non-proliferative diabetic retinopathy is characterized by abnormal permeability of retinal capillaries leading to retinal oedema, and closure of capillaries leading to retinal non-perfusion and ischaemia. Diabetic maculopathy occurs when these processes affect the macula and are therefore a threat to visual functioning. Clinically significant macular oedema (CSMO) is a term from the Early Treatment of Diabetic Retinopathy Study (ETDRS) [4] and is an evidence-based threshold for laser photocoagulation treatment.

Proliferative diabetic retinopathy occurs when retinal ischaemia is sufficiently severe to lead to the formation of new vessels. Visual loss occurs in proliferative diabetic retinopathy when these vessels bleed, or tractional retinal detachment ensues from fibrovascular proliferation. Without treatment, 50% of patients with proliferative diabetic retinopathy will become blind within 5 years [5]. Diabetic retinopathy can be graded on the basis of the clinical features. The grades of retinopathy correlate with likelihood of development of proliferative diabetic retinopathy and can be standardized by standard retinal photographs, as used in the Early Treatment of Diabetic Retinopathy Study [4]. The aim of this systematic review was to summarize findings from reliable research studies of estimates of the prevalence and incidence of diabetic retinopathy and maculopathy in African countries.

## Methods

### Data sources and search strategy

A systematic narrative review of published literature was performed according to the Preferred Reporting Items for Systematic Reviews and Meta-Analyses (PRISMA) statement [6]. Relevant studies published between 1948 and February 2011 were identified by searching, using a predefined strategy, the following electronic databases: MEDLINE (PubMed), EMBASE (OVID) and EMBASE Classic, Science Citation index and Conference Proceedings Citation index (ISI Web of Science). The following were also searched: the African regional database ‘African Index Medicus’, the grey literature database ‘OpenSIGLE’, the World Health Organization (WHO) International Clinical Trials Registry and the meta-Register of Controlled Trials (mRCT). Customized searches were developed by one of the authors (PIB) in conjunction with a Cochrane Collaboration-trained trials coordinator. Search histories are reproduced in the Supporting Information (Appendix S1). No language, publication status, time limits or language restrictions were applied to electronic searches. Search results were merged using reference management software (Endnote, Thomson Reuters) and duplicate records removed. The reference lists of articles identified, including existing reviews, were hand-searched.

### Selection criteria

The following were included: studies reporting prevalence or incidence or progression of diabetic retinopathy or diabetic maculopathy; cross-sectional or cohort study design; studies of subjects with diabetes mellitus resident in African countries. Exclusion criteria were: studies with fewer than 50 subjects; studies of populations of African origin residing outside the continent; reports not published in English; case series and conference abstracts. To improve the current relevance of the review those reports published before 1990 were excluded.

The method used to apply selection criteria was as follows. Titles and abstracts were examined by one investigator (PIB) and obviously irrelevant reports removed. Full text copies of the potentially relevant reports were retrieved. Multiple reports of the same study were linked together. Full-text reports were examined independently by two investigators (PIB and IJCM) for compliance with eligibility criteria. Disagreements were resolved by discussion.

### Data extraction and assessment of risk of bias

Major outcome variables were extracted independently by two investigators (PIB and IJCM) into a spreadsheet (Excel, Microsoft) with a standardized approach. Any disagreement was resolved by discussion. The main outcome variables extracted were the prevalence of diabetic retinopathy, proliferative diabetic retinopathy and diabetic maculopathy and the incidence of diabetic retinopathy, proliferative diabetic retinopathy and diabetic maculopathy. Prevalence of grades of retinopathy were recorded by patient according to the worse eye and, unless stated, are presented as such below. Studies were stratified by the source of the population sample (with community studies more likely to give a more accurate population-based assessment of prevalence); and risk of bias was assessed by seeking evidence of incomplete outcome data (missing data, patients excluded from report, patients lost to follow-up in cohort studies).

## Results

**Figure 1:**
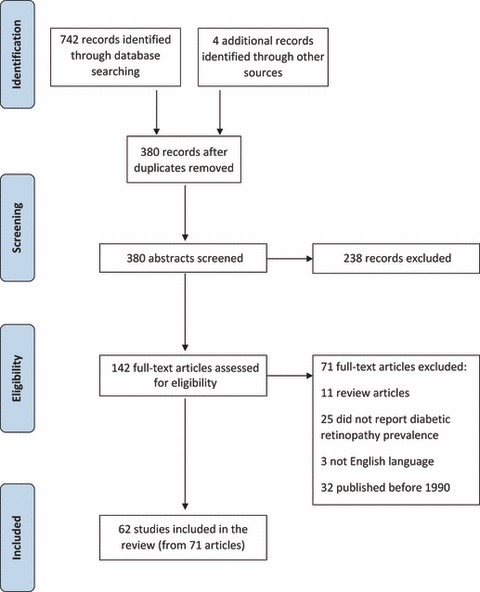
Identification process for eligible studies. Format reproduced from the PRISMA statement [6].

The literature search yielded 380 citations, of which 142 were reviewed in full text; 71 met the inclusion criteria and reported on a total of 62 studies (Fig. 1) [7–60], and see also Supporting Information (Appendix S2 [82–98]). Literature search report reproduced in the Supporting Information (Table S1).

### Characteristics of included studies

Characteristics of the included studies are summarized in the Supporting Information (Table S2).

#### Design

Only three community-based studies were identified [7–9]. In Mauritius, in 1998, researchers followed up the population-based study performed in 1992 [7] with a survey of the same cohort 6 years later [10]. An additional cohort study followed a group of patients with Type 1 diabetes identified from a hospital clinic [11–13]. All other studies were clinic-based surveys or case–control studies; the majority were undertaken in diabetes clinics (hospital or primary care) or hospital ophthalmology clinics.

#### Distribution

**Figure 2:**
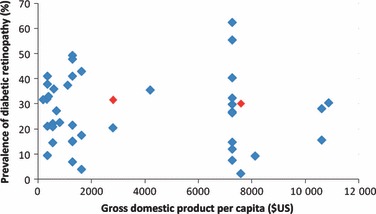
Prevalence of diabetic retinopathy in patients with diabetes according to national per capita gross domestic product. Red markers: population-based studies. Blue markers: cohort and clinic-based studies. For cohort studies prevalence in baseline survey shown. Gross domestic product per capita figures: International Monetary Fund (2011) [79].

The 62 studies were performed in 21 countries. Geographical distribution of studies was uneven and, within geographical regions, certain countries were over-represented. All of 19 studies undertaken in Western Africa took place in Nigeria, except one that covered Nigeria and Ghana [14] and one from Mali [15]. Within East Africa, two studies were conducted in the Seychelles [16,17] and two in Mauritius [7,10]: relatively wealthy, ethnically diverse, small island nations. There was no clear correlation between the average standard of living in a country, as measured by per capita gross domestic product (GDP) and reported prevalence of diabetic retinopathy (Fig. 2) or proliferative diabetic retinopathy (see also Supporting Information, Fig. S1). Only five studies specifically reported data from rural populations [7–9,18,19].

#### Patient selection

Clinic-based studies were highly heterogeneous in patient selection in relation to age range, gender, ethnicity, duration and type of diabetes and co-morbidity. Of those studies conducted in diabetes clinics, 18 included all patients with diabetes attending the clinic, while 14 confined their study to a subgroup; for example, subjects with Type 2 diabetes [20], children 5–18 years [21] or patients with duration of diabetes > 5 years [22]. Of the nine studies conducted in ophthalmology clinics, four studied patients with a particular diagnosis (neovascular glaucoma [23], retinal disease [24,25], blindness [26]), one studied patients attending specific diabetes eye clinics [17] and four studied a cross section of all eye patients [27–30]. In those studies that differentiated Type 1 and Type 2 diabetes (30 studies), most used study-specific definitions, making inter-study comparisons problematic.

#### Assessment of retinopathy

Methods of assessment and classification of retinopathy varied widely. Only nine studies used retinal photography [77,^10^,^31^–^31^] and six of these were conducted in South Africa [31–33,35–37]. Thirty studies classified retinopathy simply as present or absent; 32 used a recognized grading system. Most used an adaptation of the Early Treatment of Diabetic Retinopathy Study grading system [4]. However, the application and its reporting varied widely. In no study was an external validation of the practitioner’s grading reported.

#### Evidence of bias

There was evidence of incomplete outcome data in a number of studies. In the majority of clinic-based studies, the number of patients approached to participate was not reported, making selection bias difficult to assess. Many studies reported prevalence of a number of diabetic complications. In some studies, a low proportion of patients were examined for retinopathy. For example, in Harzallah *et al*. [38] only 19% of 593 patients underwent retinal examination. Many studies excluded patients with significant cornea or media opacities [39,40] or with ungradeable photographs [31].

### Community-based studies

We identified three community-based studies (Table 1). In Egypt, in 1991–1994, researchers examined the prevalence of diabetes and the relationship between HbA_1c_ and retinopathy [8,34,41,42]. Articles by Herman *et al*. [34] and Penman *et al*. [41] report different prevalence of diabetic retinopathy as graded from retinal photography: 35.4% in 376 subjects in the former and 31.6% in 335 subjects in the latter. No explanation for this difference between the two reports is offered, suggesting missing data in one or both analyses. Herman *et al*. [34] demonstrated in multivariate analysis that diabetic retinopathy was associated with longer duration of diabetes (per 10 years) (odds ratio = 1.37, 95% CI 1.09–1.73) and higher HbA_1c_ (per unit) (odds ratio = 1.15, 95% CI 1.03–1.27).

**Table 1:** Community-based cross-sectional studies reporting prevalence of diabetic retinopathy in Africa

Study	Methods	Subjects and subgroups	*n*	Outcome		
				Any diabetic retinopathy, % (95% CI)	Proliferative diabetic retinopathy (%)	Maculopathy (%)
Studies reporting prevalence of diabetes and diabetic retinopathy in the general population						
Egypt, 1991–1994 [8,34,41,42]	Stratified random sampling of persons ≥ 20 years in urban and rural areas near Cairo. 4620 adults underwent random glucose testing. Those at high risk of diabetes and a sample of those at low risk (total 1451) had a fasting glucose test. Diabetes diagnosed by World Health Organization criteria (see also Supporting Information, Appendix S1 [101]). Retinal photography graded according to Airlie House Classification and binocular indirect ophthalmoscopy examination by ophthalmologist. Those ungradeable on photography and binocular indirect ophthalmoscopy excluded from analysis of retinal photography and binocular indirect ophthalmoscopy, respectively	Subjects with diabetes (retinal photography)*	335	31.6	0.9	4.5‡
		Subjects with diabetes (binocular indirect ophthalmoscopy)*	404	20.3	0	1.2‡
		Known diabetes (retinal photography)†	287	41.5 (35.8–47.2)		
		Newly diagnosed diabetes (retinal photography)†	89	15.7 (8.2–23.3)		
		Impaired glucose tolerance (retinal photography)†	103	1.9 (0–4.6)		
Mauritius, 1992 [7]	6553 persons in 14 geographically defined clusters underwent glucose tolerance test. In 11 clusters, all adults aged 25–74 years were invited to attend; in three clusters, age-stratified sampling of adults aged 35–64 years performed. Those with diabetes and 25% of those with impaired glucose tolerance [World Health Organization criteria (115)] had 3-field, 45° stereoscopic retinal photography of the right eye. Grading by certified assessor according to modified Airlie House criteria. Those with ungradeable photographs were excluded from analysis	All subjects with diabetes	746	30.2 (26.9–33.5)	1.3	
		Known diabetes	388	44.3 (39.4–49.2)	2.3	
		Newly diagnosed diabetes	358	14.8 (11.1–18.5)	0.3	
		Impaired glucose tolerance	165	9.1 (4.7–13.5)	0	
		Muslim Indian race with diabetes	186	22.8 (18.7–28.9)	1.1	
		Creole race with diabetes	160	35.7 (28.1–43.3)	1.3	
Study of cause of visual impairment in the general population						
Nigeria, 2005–2007 [9]	National multistage, stratified cluster sampling of persons ≥ 40 years to determine cause of visual impairment. 13 591 visual acuity tested; 3129 had uncorrected visual acuity < 6/12 in better eye examined by ophthalmologist. Primary cause of visual impairment recorded	Subjects with visual acuity < 6/12 better eye	3129	0.29		

The prevalence of diabetic retinopathy in Egypt, 1991–1994, was reported in four publications:

* denotes data from Penman *et al*. (1998) [41]

† denotes data from Herman *et al*. (1998) [34].

‡ ‡Maculopathy in Penman *et al*. (1998) [41] defined as any exudates present in the macular region.

In 1992, researchers in Mauritius [7] investigated prevalence of and risk factors for diabetic retinopathy in Asian Indian, Chinese and Creole Mauritians. This high-quality study demonstrated a high prevalence of diabetic retinopathy in all major ethnic groups in Mauritius. The prevalence of diabetic retinopathy and proliferative diabetic retinopathy were particularly high in known diabetes: 44.3 and 2.3%, respectively. Muslim Indians had the lowest prevalence of retinopathy (10.8 and 34.0% for new and known diabetes, respectively); significantly lower than Creoles (18.8 and 53.8%, respectively). The following were independently associated with retinopathy: duration of diabetes, fasting plasma glucose, systolic blood pressure, albuminuria and decreasing BMI.

### Cohort studies

Table 2 summarizes cohort studies of diabetic retinopathy conducted in Africa. In Mauritius, in 1998, researchers followed up the population-based study performed in 1992 [7] with a survey of diabetes complications [10]. Of subjects with diabetes in the initial survey, 40.5% were re-examined. The 6-year incidence of diabetic retinopathy and proliferative diabetic retinopathy in subjects with diabetes but no diabetic retinopathy in the first survey was 23.8 and 0.4%, respectively. The incidence of proliferative diabetic retinopathy was much higher in subjects with mild non-proliferative diabetic retinopathy (5.2%) and moderate non-proliferative diabetic retinopathy (29.4%) in the first survey. Duration of diabetes and fasting blood glucose were independently associated with incidence of retinopathy. In South Africa, in 1982–2002, Gill and co-workers identified a cohort of patients with diabetes requiring insulin therapy diagnosed before age 30 years [11]. In those subjects seen at 10 years, prevalence of diabetic retinopathy had increased from 6 to 52% and proliferative diabetic retinopathy from 0 to 3% [12]. In subjects seen at 20 years, prevalence of diabetic retinopathy had increased from 12 to 59% [13].

**Table 2:** Cohort studies reporting prevalence and incidence of diabetic retinopathy in Africa

Study	Methods	Subjects and subgroups	n	Outcome		
				Any diabetic retinopathy, % (95% CI)	Proliferative diabetic retinopathy, %	Percentage of subjects progressing
1. Mauritius 1992–1998						
Initial population-based study, 1992 [7]	Population-based study of prevalence of diabetes and diabetic retinopathy: methodology outlined in Table 1	All subjects with diabetes	746	30.2 (26.9–33.5)	1.3	
		Known diabetes	388	44.3 (39.4–49.2)	2.3	
		Newly diagnosed diabetes	358	14.8 (11.1–18.5)	0.3	
Second survey, 1998 [10]	Of those assessed for complications in 1992, 528 attended the follow-up survey. Grading of retinopathy as in first assessment	Subjects with diabetes	302	33.8	3.0	25.2
		Diabetes with no diabetic retinopathy at baseline	227	23.8*	0.4†	23.8
		Diabetes, mild non-proliferative diabetic retinopathy at baseline	58		5.2†	27.7
		Diabetes, moderate non-proliferative diabetic retinopathy at baseline	17		29.4†	35.3
2. South Africa 1982–2002						
Baseline assessment, 1982 [11]	88 black South Africans with diabetes requiring insulin therapy diagnosed < 30 years attending the diabetes clinic at Baragwanath Hospital, Soweto were screened for diabetic complications. 66 were examined for retinopathy by a physician using direct ophthalmoscope	Subjects with diabetes requiring insulin therapy diagnosed < 30 years	66	12.1	0	
		Subgroup subsequently seen at 10 years	33	6	0	
		Subgroup subsequently seen at 20 years	17	12		
10-year follow-up, 1992 [12]	Of the original cohort, 24 were lost to follow-up, 10 had died. Of 54 still attending clinic, 36 were examined. In three patients cataracts prevented fundal view	Subjects with diabetes requiring insulin therapy diagnosed < 30 years	33	52	3	
20-year follow-up, 2002 [13]	Of the original cohort, 21 died, 39 were lost to follow-up, 28 were still attending clinic, of which 17 were assessed for complications	Subjects with diabetes requiring insulin therapy diagnosed < 30 years	17	59		

* Incidence of diabetic retinopathy at 6 years.

† Incidence of proliferative diabetic retinopathy at 6 years.

No other prospective cohort studies were identified. However, studies reflecting cumulative incidence of diabetic retinopathy are available. In South Africa, Distiller *et al*. [32] reported on 1520 patients with Type 1 diabetes and 8026 patients with Type 2 who had maintained membership for ≥ 5 years of a community-based, privately funded diabetes management programme. In subjects with Type 1 diabetes, prevalence of any retinopathy at baseline and at 5 years was 22.3 and 28%, respectively, and in subjects with Type 2 diabetes was 20.5 and 26.6%, respectively. In retrospective studies of patients with diabetes of long duration, Lester [43] showed a prevalence of diabetic retinopathy of 45.5% in 121 Ethiopian patients with duration of diabetes > 20 years, while Distiller *et al*. [33] reported presence of diabetic retinopathy in 14.8% of 148 South African Caucasian patients with Type 1 diabetes of > 18 years duration.

### Hospital-based and primary care-based surveys

Tables 3,4 and 5 summarize hospital-based and primary care-based surveys reporting prevalence of diabetic retinopathy using a recognized grading system. The most recent large study from Northern Africa was conducted in Cairo during 2007–2008 in endocrinology clinics in two major teaching hospitals [39]. Prevalence of proliferative diabetic retinopathy (2.3%) and clinically significant macular oedema (11.5%) reported in this study was high. Of four studies from Western Africa [44–47], none reported the prevalence of maculopathy (Table 3). Only three were identified from Middle Africa [22,48,49]. Longo-Mbenza *et al*. [48] studied 3010 patients with diabetes attending diabetes primary care facilities using retinal photography; prevalence of diabetic retinopathy was 31.6%.

**Table 3:** Hospital-based surveys of patients with diabetes reporting prevalence of diabetic retinopathy using a recognized grading system in Northern, Western and Middle Africa

Study	Country	Methods	*n*	Any diabetic retinopathy (%)	Proliferative diabetic retinopathy (%)	Clinically significant macular oedema (%)	Statistically significant associations of diabetic retinopathy
Northern Africa							
Elbagir *et al*., 1995 [53]	Sudan	Patients with diabetes requiring insulin (duration > 1 year) aged 15–75 years attending medical outpatient department examined with direct ophthalmoscope by a physician	91	43	10*	Not reported	Current age of patient, duration of diabetes, systolic blood pressure, diastolic blood pressure, cholesterol, BMI (univariate analysis)
Macky *et al*., 2011 [39]	Egypt	Patients > 18 years of age attending a diabetes clinic examined with slit-lamp biomicroscopy by ophthalmologist. Excluded 47 patients because of media opacities	1325	20.5	2.3	11.5	Duration of diabetes, hypertension, female gender (univariate analysis)
Western Africa							
Ikem and Akinola, 2001 [47]	Nigeria	Consecutive patients with Type 2 diabetes seen at medical outpatient department. Examined by physician; instrument not stated	132	41.1	1.0	Not reported	Hypertension
Alebiosu *et al*., 2003 [44]	Nigeria	Hospitalized subjects with Type 2 diabetes and nephropathy. Examined with direct ophthalmoscope by physician	191	47.1	12.6	Not reported	Not reported
Omolase *et al*., 2010 [45]	Nigeria	Patients with diabetes attending medical outpatient department. Examined with direct ophthalmoscope by ophthalmologist	100	15.0	2.0	Not reported	Duration of diabetes (univariate analysis)
Onakpoya *et al*., 2010 [46]	Nigeria	Patients with Type 2 diabetes attending a 3° centre diabetes clinic; invited for screening by ophthalmologist with direct ophthalmoscope. 3.6% no fundal view	80	21.6	1.2	Not reported	Not reported
Middle Africa							
Sobngwi *et al*., 1999 [49]	Cameroon	Adults attending diabetes clinic. Excluded patients with renal disease. Slit lamp biomicroscopy examination by ophthalmologist	64	37.5	1.6	Not reported	Univariate analysis: current age of patient, systolic blood pressure, microalbuminuria Multivariate analysis: micro-albuminuira

* ’Severe retinopathy’ by World Health Organization multinational study criteria [80].

**Table 4:** Hospital-based surveys of patients with diabetes reporting prevalence of diabetic retinopathy using a recognized grading system in Eastern Africa

Study	Country	Methods	*n*	Any diabetic retinopathy (%)	Proliferative diabetic retinopathy (%)	Any maculopathy (%)	Statistically significant associations of diabetic retinopathy
Sulivan *et al*., 1990 [16]	Seychelles	Patients with diabetes requiring insulin therapy attending diabetic clinic examined by a physician. Instrument not reported	108	15.7	2.8	Not reported	Not reported
Lester, 1992§	Ethiopia	Type 1 diabetes seen 1976–1990. Examination by physician. Instrument not reported	431	9.5	2.6	1.2	Not reported
Lester 1993§	Ethiopia	Type 2 diabetes seen 1976–1991. Examination by physician. Instrument not reported	503	41.1	6.9	4.0	Not reported
Taylor *et al*., 1997 [17]	Seychelles	Type 2 diabetes: 184 attending an eye clinic, 199 invited for screening. Ophthalmologist slit-lamp biomicroscopy examination	383	28	4	19	Insulin therapy, duration
Seyoum and Mengistu, 2001 [54]	Ethiopia	Patients attending a diabetes clinic. Direct ophthalmoscope examination by ophthalmologist. Three patients excludedas no fundal view	302	37.8	1.7	Not reported	Current age of patient, duration of diabetes, systolic blood pressure, diastolic blood pressure, proteinuria
Teshome and Melaku, 2004 [25]	Ethiopia	Consecutive patients seen at a retinal clinic (not all had diabetes). Slit-lamp biomicroscopy examination by ophthalmologist	1390	28.7	9.9	11.1‡	Not reported
Mumba *et al.*, 2007 [55]	Tanzania	Patients > 18 years attending diabetes clinic. No previous fundus examination. Slit-lamp biomicroscopy examination by ophthalmologist	86	20.9	1.2	Not reported	Not reported
Mwale *et al*., 2007 [40]	Kenya	Clinic patients with Type 2 diabetes. Slit-lamp biomicroscopy examination by ophthalmologist. Excluded cornea or media opacity	96	22.6	0	Not reported	Not reported
Gill *et al*., 2008 [18]	Ethiopia	Consecutive patients attending hospital diabetes clinic in a remote region. Slit-lamp biomicroscopy examination by ophthalmologist	105	21	1.9	Not reported	Not reported
Glover *et al*., 2011 [52]	Malawi	Consecutive adults attending a hospital diabetes clinic. Slit-lamp biomicroscopy examination by ophthalmologist	281	32.0	5.7	15.0*	Albuminuria, neuropathy, insulin therapy†

* Sight-threatening maculopathy according to Liverpool Diabetic Eye Study adaptation of the Early Treatment Diabetic Retinopathy Study (ETDRS) grading [81].

† Multivariate associations of sight-threatening diabetic retinopathy for patients with Type 2 diabetes.

‡ Clinically significant macular oedema.

§ Multiple publications [43,56,57] with overlapping populations have emanated from the diabetes clinic at Yekatit 12 Hospital, Addis Ababa, Ethiopia. For the purposes of this review, these papers are viewed as one study. Data presented from Lester 1992 [56] and Lester 1993 [57].

**Table 5:** Hospital-based and primary care-based surveys of patients with diabetes reporting prevalence of diabetic retinopathy using a recognized grading system in Southern Africa

Study	Country	Methods	*n*	Any diabetic retinopathy (%)	Proliferative diabetic retinopathy (%)	Clinically significant macular oedema (%)	Statistically significant associations of diabetic retinopathy
Mollentz *et al*., 1990 [37]	South Africa	Black patients with diabetes > 5 years duration attending diabetes clinic. Retinal photography graded by ophthalmologist	86	29.7‡	1.2‡	Not reported	Not reported
Levitt *et al*., 1997 [50]	South Africa	Black Africans attending diabetes primary care service. Examined by a physician with direct ophthalmoscope	243	55.4	4.3	31.1†	Not reported
Rotchford and Rotchford, 2002 [19]	South Africa	Adults attending a nurse-led primary care diabetes service in rural KwaZulu-Natal. Examined with slit-lamp biomicroscopy by an ophthalmologist	253	40.3	5.6	10.3	Albuminuria, duration of diabetes, current age of patient, HbA_1c_, BMI (inverse)
Huddle, 2005 [58]	South Africa	Pregnant women with diabetes attending a clinic: Type 1, Type 2 and gestational diabetes. Direct ophthalmoscope examination; practitioner grading retinopathy not reported	733	7.6	0.1	Not reported	Type 1 diabetes
Carmichael *et al*., 2005* [31]	South Africa	Patients attending an urban diabetes clinic: 588 black, 739 white, 180 indian. Retinal photography graded by ophthalmologist. Ungradeable photographs excluded	1517	26.5	Not reported	Not reported	Duration of diabetes, insulin therapy, albumin–creatinine ratio, systolic blood pressure
Mengesha, 2006 [59]	Botswana	Patients with diabetes attending government health facilities. Slit-lamp biomicroscopy examination by ophthalmologist	401	9.2	3.0	Not reported	Not reported
Mash *et al*., 2007 [36]	South Africa	Patients attending primary care diabetes service: 44%‘black’; 56%‘coloured’. Retinal photography graded by ophthalmologist. 17.5% of photographs ungradeable	400	62.4	6.1	15.2†	Not reported
Read and Cook, 2007 [51]	South Africa	Patients with Type 2 diabetes attending a primary care diabetes clinic (124 ‘Black’; 119 ‘Coloured’; 5 ‘White’; 1 ‘Asian’). Direct ophthalmoscope examination by ophthalmologist	248	32.3	2.4	8.5	Not reported

* Three reports [31,35,60] described grades of diabetic retinopathy in overlapping populations. Figure for any diabetic retinopathy taken from the largest report [31] (*n* = 1517); associations of diabetic retinopathy taken from smaller report (*n* = 507) [35].

† Any maculopathy.

‡ ‡Percentage of eyes (not patients) with specified grade of diabetic retinopathy.

Hospital-based surveys from Eastern Africa cover nine countries showing a general trend of increasing prevalence of diabetic retinopathy from earlier to more recent studies (Table 4). Diabetes clinic-based surveys from Southern Africa in general report higher prevalence of diabetic retinopathy and proliferative diabetic retinopathy than comparable clinics in other regions of Africa (Table 5). Proliferative diabetic retinopathy prevalence > 4% was recorded in three studies of unselected diabetes clinic attendees from South Africa [19,36,50]. Data on prevalence of diabetic maculopathy were limited from all regions. However, eight studies suggest high prevalence [17,19,25,36,39,50–52]. Of note, three South African, primary care-based studies were identified. Levitt *et al*. [50], Mash *et al*. [36] and Read and Cook [51] reported high prevalence of proliferative diabetic retinopathy and maculopathy (Table 5), comparable with hospital-based surveys in the same country and higher than hospital-based surveys elsewhere in Africa.

Two studies from South Africa compared prevalence of diabetic retinopathy in different ethnic groups [35,51]. The authors acknowledge the effect of environmental factors on different racial communities, even in the post-apartheid era. Kalk *et al*. [35] studied 507 ‘poor or indigent’ patients attending a free hospital diabetes clinic. Prevalence of diabetic retinopathy was similar in patients of African (37%), European (41%) or Indian (37%) heritage. However, ‘severe diabetic retinopathy’ (study-specific classification) was significantly more frequent in Africans (52%) and Indians (41%) compared with Europeans (26%). Read and Cook [51] found no relationship between ethnicity and diabetic retinopathy prevalence.

### Studies reporting visual acuity

Nineteen studies reported visual acuity in subjects with diabetes; parameters reported varied widely between studies. Only the Nigerian national blindness and visual impairment survey [9] tested logarithm of the minimum angle of resolution (logMAR) acuity. The population-based Mauritius diabetes complication study [7] reported best-corrected visual acuity < 6/12 in 7.1% of subjects with diabetes at baseline. There was no difference in this figure for subjects with and without retinopathy. The Diabetes in Egypt project [41] reported visual acuity in 427 subjects with diabetes. Of these, 31 (7.3%) were blind (defined as best-corrected visual acuity in the better eye less than 6/60); 239 (56%) had a best-corrected visual acuity between 6/12 and 6/60. It is likely that media opacities accounted for a proportion of this visual impairment: 123 eyes had cataract; 11 had corneal opacity; 17 had both.

The Nigerian national blindness and visual impairment survey was conducted between 2005 and 2007 [9]. Diabetic retinopathy was identified as the primary cause of visual impairment in 0.29% of 3129 subjects with uncorrected visual acuity worse than 6/12 and in 0.5% of those with acuity less than 3/60. This study is likely to underestimate the visual impact of diabetic retinopathy as examiners were instructed to preferentially record treatable, then preventable causes of visual impairment; i.e. cataract would be recorded in preference to diabetic retinopathy if both were affecting visual acuity to similar degrees.

## Discussion

This systematic narrative review describes 62 studies reporting the prevalence and incidence of diabetic retinopathy and maculopathy in Africa. The methodological approach used standard inclusion, appraisal and data extraction approaches. Few high-quality, population-based studies were identified: the majority of studies were surveys of hospital clinic attendees. Identified studies were highly heterogeneous in terms of patient selection and method of assessment and classification of retinopathy. Despite these inconsistencies between studies, the review identified rates of prevalence of diabetic retinopathy in many areas of Africa comparable with high-income countries. Prevalence of proliferative diabetic retinopathy and maculopathy was high in recent studies, particularly those from Southern and Eastern Africa. Common themes were identified in the associations of diabetic retinopathy and impact on vision.

### Methodology of included studies

The review identified three high-quality, population-based, cross-sectional studies of diabetic retinopathy epidemiology [7–9]. Only two cohort studies were identified. Large epidemiological studies are expensive; the population-based studies were conducted in states with relatively greater resources: Nigeria, Mauritius and Egypt. The lack of studies from Middle Africa is likely to reflect lack of resources, poor health infrastructure and deficiency of trained medical professionals. The relatively small number of studies identified from Northern Africa is partially explained by the tendency of francophone countries to publish in French.

The literature is dominated by studies of urban populations reflecting the distribution of major health facilities. Urbanization is seen as an important factor driving the diabetes epidemic [61]; studies of urban populations may overestimate diabetic retinopathy prevalence. A caveat is that, in resource-poor settings, patients travel long distances to health facilities and rural patients may therefore be included. The majority of studies identified were hospital clinic-based surveys; selection bias is a major issue and the findings should be generalized to other settings with caution. Another bias is that clinics are seen by many as a point to collect medication; patients with diet-controlled diabetes may be under-represented. The classification of diabetes in Africa is problematic, particularly where investigations are limited. Disease characteristics differ from Caucasian populations. For example, peak age of onset of Type 1 diabetes is later in African communities, typically 22–29 years [62]. Other phenotypes of diabetes are recognized in patients of African origin, including ‘atypical African diabetes’ and ‘malnutrition-related diabetes’ [63].

Adaptations of the Early Treatment of Diabetic Retinopathy Study grading system have become the accepted reference standard for classifying retinopathy in research settings. Despite this, its use in everyday clinical practice is difficult because of a large number of levels requiring correlations with standard photographs and grading rules that must be remembered. General ophthalmologists and physicians in resource-poor settings may not be able to use this system to a reproducible level. Stereoscopic photography with validated grading is rapidly becoming the reference standard for assessing retinopathy. Digital photography allows transfer of images to distant reading centres, as was used in the Diabetes in Egypt project [8,34,41,42]. While expensive, this may be the direction of future research.

### Prevalence and incidence of diabetic retinopathy and diabetic maculopathy

Community-based studies identified in this review reported prevalence rates of diabetic retinopathy and proliferative diabetic retinopathy comparable with American and European populations with diabetes. The Diabetes in Egypt project [8,34,41,42] reported a prevalence of diabetic retinopathy and proliferative diabetic retinopathy in subjects with diabetes of 31.6 and 0.9%, respectively. The Mauritius diabetes complication study [7] reported 30.2% diabetic retinopathy and 1.3% proliferative diabetic retinopathy; the prevalence of proliferative diabetic retinopathy in subjects with known diabetes was 2.3%. In comparison, a 2005–2008 cross-sectional sample of US adults with diabetes aged 40 years and older estimated prevalence of diabetic retinopathy and proliferative diabetic retinopathy as 28.5% and 1.5%, respectively [64]. Recent population-based studies in Europe have reported similar rates [65–69]. Younis *et al.* [65] studied 8062 patients with diabetes entering an English primary care-based screening programme. The prevalences of any retinopathy and proliferative diabetic retinopathy in Type 1 diabetes were 45.7 and 3.7%, respectively, and in Type 2 diabetes were 25.3 and 0.5%, respectively.

The lack of community-based studies from Sub-Saharan Africa is important. Very high prevalence of diabetic retinopathy, proliferative diabetic retinopathy and maculopathy has been reported in notable high-quality, clinic-based surveys in the last decade: in Eastern Africa by Glover *et al*. [52] (32.0% diabetic retinopathy, 5.7% proliferative diabetic retinopathy, 15% sight-threatening maculopathy), and in South Africa by Mash *et al*. [36] (62.4% diabetic retinopathy, 6.1% proliferative diabetic retinopathy, 15.2% any maculopathy) and Rotchford and Rotchford [19] (40.3% diabetic retinopathy, 5.6% proliferative diabetic retinopathy, 10.3% clinically significant macular oedema). These figures are likely to reflect factors including ethnicity, poor access to medical services, late diagnosis, and co-pathology including infection (importantly HIV and malaria), hypertension, malnutrition, and anaemia. We found no clear relationship between per capita gross domestic product and prevalence of diabetic retinopathy or proliferative diabetic retinopathy. However, the increased infrastructure to detect disease in states with greater resources is an important confounding factor.

The influence of ethnicity on diabetic retinopathy prevalence in populations of African origin has yet to be determined. In the USA, Zhang *et al*. [64] reported prevalence of both diabetic retinopathy and vision-threatening retinopathy (defined as Early Treatment of Diabetic Retinopathy Study severe non-proliferative diabetic retinopathy, proliferative diabetic retinopathy, or clinically significant macular oedema) to be higher in non-Hispanic black subjects (38.8 and 9.3%, respectively) compared with non-Hispanic white subjects (26.4 and 3.2%, respectively). Previous studies have shown similar results [70,71]. However, differences were attributable to risk factors for retinopathy [71]. Therefore, while associations between polymorphisms of specific genes and diabetic retinopathy have been described in African populations [72], no ethnic propensity to retinopathy has been identified.

Neither of the two cohort studies identified by this review reported two- or three-step progression on the Early Treatment of Diabetic Retinopathy Study scale, as used in recent European studies [73]. The Mauritius diabetes complication study [10] reported 6-year incidence of diabetic retinopathy (23.8%). Six-year progression to proliferative diabetic retinopathy was reported from no diabetic retinopathy (0.4%), mild non-proliferative diabetic retinopathy (5.2%) and moderate non-proliferative diabetic retinopathy (29.4%). The UK Prospective Diabetes Study (UKPDS) reported similar 6-year incidence of diabetic retinopathy: 22% [74]. However, the UKPDS population were studied from a later time point: clinical diagnosis of diabetes. In the Wisconsin Epidemiological Study of Diabetic Retinopathy (WESDR) 4-year progression of diabetic retinopathy and progression to proliferative diabetic retinopathy was observed in 41.2 and 10.5% of subjects with Type I diabetes, 34 and 7.4% of insulin-treated patients with Type 2 diabetes and 24.9 and 2.3% of non-insulin treated patients, respectively [75].

### Impact of diabetic retinopathy on vision

Estimates of the proportion of African patients with diabetes who are visually impaired are high even compared with older European and American studies. Of subjects in the Diabetes in Egypt project [41], 7.3% had best-corrected visual acuity in the better eye < 6/60. In contrast, of the population in the Wisconsin Epidemiological Study of Diabetic Retinopathy, 3.6% of patients aged < 30 years at diagnosis, and 1.6% of patients aged ≥ 30 years at diagnosis were legally blind according to US standards [76,77]. The World Health Organization estimates that, in the USA and Canada, 17% of blindness is attributable to diabetic retinopathy [78]. While data are sparse, the proportion of visual impairment and blindness as a result of diabetic retinopathy in Africa appears to be considerably less. However, the prevalence of visual impairment and blindness is significantly higher in Africa [78], reflecting high prevalence of pathologies including uncorrected refractive error, cataract, corneal opacities and glaucoma.

The findings of this review have important implications for both research and clinical practice. Large, community-based cross-sectional and cohort studies are needed to investigate rates and determinants of diabetic retinopathy prevalence, incidence and progression across Africa. Consensus is needed on standardized data sets and the most appropriate methods of identification and classification of diabetic retinopathy in Africa. In Europe and America, there is strong evidence for the role of poor glycaemic control and co-pathology, including hypertension in development and progression of diabetic retinopathy. Similarly, a strong evidence base exists for the treatment of sight-threatening diabetic retinopathy with laser photo-coagulation and intravitreal agents. This evidence has yet to be accrued in African settings. Management of systemic disease and screening and treatment of retinopathy requires substantial infrastructure, which is currently lacking in many African states. The public health and health economic challenges for policymakers across Africa are significant.

## Funding sources

None.

## Competing interests

Nothing to declare.

## Supporting Information

**Figure S1.** Prevalence of proliferative diabetic retinopathy in patients with diabetes according to national per capita gross domestic product. **Table S1.** Literature search report for articles reporting prevalence, incidence or progression of diabetic retinopathy or diabetic maculopathy in African countries. **Table S2.** Characteristics of 62 studies reporting prevalence of diabetic retinopathy and maculopathy in Africa. **Appendix S1.** Search histories. **Appendix S2.** Supplementary references.
