# Supplementary material for: Epidemiology of diabetic retinopathy and maculopathy in Africa: a systematic review
Source: Diabet Med. 2013 Apr;30(4):399–412. doi: 10.1111/j.1464-5491.2012.03756.x (PMC4463765; doi:10.1111/j.1464-5491.2012.03756.x)
Supplement: Supplementary file 1 — Figure S1. Prevalence of proliferative diabetic retinopathy in patients with diabetes according to national per capita gross domestic product. Table S1. Literature search report for articles reporting prevalence, incidence or progression of diabetic retinopathy or diabetic maculopathy in African countries. Table S2. Characteristics of 62 studies reporting prevalence of diabetic retinopathy and maculopathy in Africa. Appendix S1. Search histories. Appendix S2. Supplementary references. [file dme0030-0399-sd1.doc]

**ONLINE SUPPORTING MATERIAL**

**SEARCH HISTORIES**

Search histories for the electronic databases Medline (PubMed), EMBASE, Web of Science, African index Medicus and OpenSigle. African countries search terms reproduced from [100].

**Search History Pubmed:**

#1 Search "Diabetic Retinopathy"[Mesh]

#2 Search Diabetic maculopathy ti, ab

#3 Search diabet* AND ((macular edema) OR (macular oedema))

#4 Search #1 OR #2 OR #3

#5 Search ("Africa"[MeSH] OR Africa*[tw] OR Algeria[tw] OR Angola[tw] OR Benin[tw] OR Botswana[tw] OR "Burkina Faso"[tw] OR Burundi[tw] OR Cameroon[tw] OR "Canary Islands"[tw] OR "Cape Verde"[tw] OR "Central African Republic"[tw] OR Chad[tw] OR Comoros[tw] OR Congo[tw] OR "Democratic Republic of Congo"[tw] OR Djibouti[tw] OR Egypt[tw] OR "Equatorial Guinea"[tw] OR Eritrea[tw] OR Ethiopia[tw] OR Gabon[tw] OR Gambia[tw] OR Ghana[tw] OR Guinea[tw] OR "Guinea Bissau"[tw] OR "Ivory Coast"[tw] OR "Cote d’Ivoire"[tw] OR Jamahiriya[tw] OR Jamahiryia[tw] OR Kenya[tw] OR Lesotho[tw] OR Liberia[tw] OR Libya[tw] OR Libia[tw] OR Madagascar[tw] OR Malawi[tw] OR Mali[tw] OR Mauritania[tw] OR Mauritius[tw] OR Mayote[tw] OR Morocco[tw] OR Mozambique[tw] OR Mocambique[tw] OR Namibia[tw] OR Niger[tw] OR Nigeria[tw] OR Principe[tw] OR Reunion[tw] OR Rwanda[tw] OR "Sao Tome"[tw] OR Senegal[tw] OR Seychelles[tw] OR "Sierra Leone"[tw] OR Somalia[tw] OR "South Africa"[tw] OR "St Helena"[tw] OR Sudan[tw] OR Swaziland[tw] OR Tanzania[tw] OR Togo[tw] OR Tunisia[tw] OR Uganda[tw] OR "Western Sahara"[tw] OR Zaire[tw] OR Zambia[tw] OR Zimbabwe[tw] OR "Central Africa"[tw] OR "Central African"[tw] OR "West Africa"[tw] OR "West African"[tw] OR "Western Africa"[tw] OR "Western African"[tw] OR "East Africa"[tw] OR "East African"[tw] OR "Eastern Africa"[tw] OR "Eastern African"[tw] OR "North Africa"[tw] OR "North African"[tw] OR "Northern Africa"[tw] OR "Northern African"[tw] OR "South African"[tw] OR "Southern Africa"[tw] OR "Southern African"[tw] OR "sub Saharan Africa"[tw] OR "sub Saharan African"[tw] OR "subSaharan Africa"[tw] OR "subSaharan African"[tw]) NOT ("guinea pig"[tw] OR "guinea pigs"[tw] OR "aspergillus niger"[tw])

#6 Search #4 And #5

#7 Limit #6 to Human

**Search history EMBASE:**

1 diabetic retinopathy.mp. or diabetic retinopathy/

2 diabetic macular edema/ or retina maculopathy/ or retina macula edema/ or diabetic maculopathy.mp.

3 (diabet* and (macul* edema or macul* oedema)).mp. [mp=title, abstract, subject headings, heading word, drug trade name, original title, device manufacturer, drug manufacturer]

4 (diabet* and macul*).mp. [mp=title, abstract, subject headings, heading word, drug trade name, original title, device manufacturer, drug manufacturer]

5 1 or 2 or 3 or 4

6 (Africa or Africa* or Algeria or Angola or Benin or Botswana or Burkina Faso or Burundi or Cameroon or Canary Islands or Cape Verde or Central African Republic or Chad or Comoros or Congo or Democratic Republic of Congo or Djibouti or Egypt or Equatorial Guinea or Eritrea or Ethiopia or Gabon or Gambia or Ghana or Guinea or Guinea Bissau or Ivory Coast or (Cote and Ivoire) or Jamahiriya or Jamahiryia or Kenya or Lesotho or Liberia or Libya or Libia or Madagascar or Malawi or Mali or Mauritania or Mauritius or Mayote or Morocco or Mozambique or Mocambique or Namibia or Niger or Nigeria or Principe or Reunion or Rwanda or Sao Tome or Senegal or Seychelles or Sierra Leone or Somalia or South Africa or St Helena or Sudan or Swaziland or Tanzania or Togo or Tunisia or Uganda or Western Sahara or Zaire or Zambia or Zimbabwe or Central Africa or Central African or West Africa or West African or Western Africa or Western African or East Africa or East African or Eastern Africa or Eastern African or North Africa or North African or Northern Africa or Northern African or South African or Southern Africa or Southern African or South Africa).mp. [mp=title, abstract, subject headings, heading word, drug trade name, original title, device manufacturer, drug manufacturer]

7 5 and 6

8 Limit 7 to Humans

**Search history Web of Science:**

(Diabetic retinopathy) or (Diabetic maculopathy) or ((diabet* AND ((macular edema) OR (macular oedema)) [Topic]

AND

(Africa or Africa* or Algeria or Angola or Benin or Botswana or Burkina Faso or Burundi or Cameroon or Canary Islands or Cape Verde or Central African Republic or Chad or Comoros or Congo or Democratic Republic of Congo or Djibouti or Egypt or Equatorial Guinea or Eritrea or Ethiopia or Gabon or Gambia or Ghana or Guinea or Guinea Bissau or Ivory Coast or (Cote and Ivoire) or Jamahiriya or Jamahiryia or Kenya or Lesotho or Liberia or Libya or Libia or Madagascar or Malawi or Mali or Mauritania or Mauritius or Mayote or Morocco or Mozambique or Mocambique or Namibia or Niger or Nigeria or Principe or Reunion or Rwanda or Sao Tome or Senegal or Seychelles or Sierra Leone or Somalia or South Africa or St Helena or Sudan or Swaziland or Tanzania or Togo or Tunisia or Uganda or Western Sahara or Zaire or Zambia or Zimbabwe or Central Africa or Central African or West Africa or West African or Western Africa or Western African or East Africa or East African or Eastern Africa or Eastern African or North Africa or North African or Northern Africa or Northern African or South African or Southern Africa or Southern African or South Africa) [topic]

**Search history African index Medicus, OpenSigle:**

(Diabetic retinopathy) or (Diabetic maculopathy) or ((diabet* AND ((macular edema) OR (macular oedema))

**SUPLIMENTARY TABLES AND FIGURES**

**Online table 1.** Literature search report for articles reporting prevalence, incidence or progression of DR or diabetic maculopathy in African countries. Four further articles were identified through hand searching and personal communication.

| Source | Date Range Searched | Hits Retrieved (before duplicate removal) |
| --- | --- | --- |
| **ELECTRONIC DATABASES** |  |  |
| Medline (Pubmed) | 1948 – 6/2/2011 | 204 |
| Embase (OVID) + Embase Classic | 1947 – 6/2/2011 | 333 |
| Science Citation index + Conference Proceedings Citation index (ISI Web of Science) | 1900 - 7/2/2011 | 199 |
| **Final number of records in Endnote database after deleting duplicates** |  | **370** |
|  |  |  |
| **OTHER DATABASES** |  |  |
| African Index Medicus database | Searched 8/2/2011 | 5 |
| Opensigle | Searched 8/2/2011 | 0 |
|  |  |  |
| **ONGOING TRIALS REGISTERS** |  |  |
| WHO International Clinical trials Registry  Current Controlled trials: Meta Register of Controlled trials (mRCT) | Searched 8/2/2011 | 1 |

**Online table 2. Characteristics of 62 studies reporting prevalence of diabetic retinopathy and maculopathy in Africa.**

| **Characteristic** | **Geographical region** | | | | | |
| --- | --- | --- | --- | --- | --- | --- |
|  | Northern Africa | Western Africa | Southern Africa | Middle Africa | Eastern Africa† | Total no. of studies |
| **Study design** | | | | | | |
| Community-based cross-sectional | 1 | 1 |  |  | 1* | 3 |
| Cohort |  |  | 1 |  | 1* | 2 |
| Diabetic clinic survey‡ | 4 | 5 | 10 | 2 | 11 | 32 |
| Hosp' eye clinic survey |  | 5 |  | 1 | 3 | 9 |
| Other hospital-based survey | 2 | 5 | 2 |  | 2 | 11 |
| Case control | 2 | 3 |  |  |  | 5 |
| **Year published** | | | | | | |
| 1990-1999 | 5 | 3 | 4 | 2 | 5 | 19 |
| 2000-2011 | 4 | 16 | 9 | 1 | 13 | 43 |
| **Type of diabetes** | | | | | | |
| Type 1 alone | 1 |  | 2 |  | 1 | 4 |
| Type 2 alone | 4 | 8 | 1 |  | 2 | 15 |
| Type 1 & 2 reported separately | 1 | 2 | 3 |  | 5 | 11 |
| Mixed | 3 | 9 | 7 | 3 | 10 | 32 |
| **Practitioner grading retinopathy** | | | | | | |
| Ophthalmologist | 1 | 12 | 7 | 3 | 11 | 34 |
| Physician | 4 | 3 | 2 |  | 2 | 11 |
| Trained grader | 1 |  |  |  | 2 | 3 |
| Not specified | 3 | 4 | 4 |  | 3 | 14 |
| **Instrument** | | | | | | |
| Slit lamp bio-microscopy | 2 | 7 | 3 | 2 | 9 | 23 |
| Retinal photography | 1 |  | 6 | 1 | 2 | 10 |
| Direct ophth'scope | 1 | 5 | 4 |  | 1 | 11 |
| Not specified or other | 5 | 7 |  |  | 6 | 18 |
| **Other study characteristics** | | | | | | |
| Recognised grading system used | 4 | 5 | 10 | 2 | 11 | 32 |
| Associations of DR reported | 6 | 8 | 8 | 2 | 9 | 32 |
| Vision reported | 1 | 7 | 3 | 1 | 7 | 19 |
| **Total number of studies** | **9** | **19** | **13** | **3** | **18** |  |

Geographical region defined according to UN scheme of geographical regions [99]. * Initial population based survey performed in Mauritius in 1992[7] followed up with a survey of the same cohort 6 years later [10]. † Includes 2 studies from the Seychelles and 1 from Mauritius. ‡ Hospital or primary care diabetes clinic.

**Online figure 1.** Prevalence of PDR in patients with diabetes according to national per capita gross domestic product (GDP). Red markers: population-based studies. Blue markers: cohort and clinic-based studies. For cohort studies prevalence in baseline survey shown.


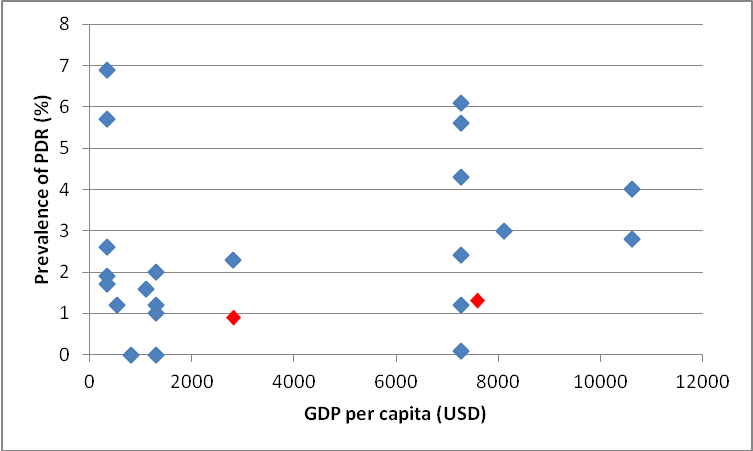


**SUPPLEMENTARY REFERENCES**

82. Akanji AO, Bella AF, Osotimehin BO. Cheiroarthropathy and long term diabetic complications in Nigerians. Ann Rheum Dis. 1990 Jan;49(1):28-30.

83. Babalola RO, Ajayi AA. A Cross-Sectional Study of Echocardiographic Indexes, Treadmill Exercise Capacity and Microvascular Complications in Nigerian Patients with Hypertension Associated with Diabetes-Mellitus. Diabetic Medicine. 1992 Dec;9(10):899-903.

84. Danbauchi SS, Anumah FE, Alhassan MA, David SO, Onyemelukwe GC, Oyati IA. Left ventricular function in type 2 diabetes patients without cardiac symptoms in Zaria, Nigeria. Ethnicity & Disease. 2005 Fal;15(4):635-40.

85. Elamin A, Altahir H, Ismail B, Tuvemo T. Clinical pattern of childhood type 1 (insulin-dependent) diabetes mellitus in the Sudan. Diabetologia. 1992 Jul;35(7):645-8.

86. Erasmus RT, Alanamu RA, Bojuwoye B. Plasma lipids and lipoproteins in type II diabetic Nigerians with retinopathy. Trop Geogr Med. 1991 Jan-Apr;43(1-2):55-8.

87. Erasmus RT, Oyeyinka G, Arije A. Microalbuminuria in non-insulin-dependent (type 2) Nigerian diabetics: Relation to glycaemic control, blood pressure and retinopathy. Postgraduate Medical Journal. 1992;68 (802):638-42.

88. Ezzidi I, Mtiraoui N, Chaieb M, Kacem M, Mahjoub T, Almawi WY. Diabetic retinopathy, PAI-1 4G/5G and -844G/A polymorphisms, and changes in circulating PAI-1 levels in Tunisian type 2 diabetes patients. Diabetes Metab. 2009 Jun;35(3):214-9.

89. Longo-Mbenza B, Muaka MM, Mbenza G, Mbungu-Fuele S, Mabwa-Mbalanda L, Nzuzi-Babeki V, et al. Risk factors of poor control of HBA1c and diabetic retinopathy: Paradox with insulin therapy and high values of HDL in African diabetic patients. International Journal of Diabetes and Metabolism. 2008;16 (2):69-78.

90. Neuhann HF, Warter-Neuhann C, Lyaruu I, Msuya L. Diabetes care in Kilimanjaro region: clinical presentation and problems of patients of the diabetes clinic at the regional referral hospital - an inventory before structured intervention. Diabetic Medicine. 2002 Jun;19(6):509-13.

91. Nwosu SN. Low vision in Nigerians with diabetes mellitus. Doc Ophthalmol. 2000 Jul;101(1):51-7.

92. Otiti-Sengeri J, Colebunders R, Kempen JH, Ronald A, Sande M, Katabira E. The prevalence and causes of visual loss among HIV-infected individuals in uganda. Journal of Acquired Immune Deficiency Syndromes. 2010; 53 (1):95-101.

93. Potluri R, Purmah Y, Dowlut M, Sewpaul N, Lavu D. Microvascular diabetic complications are more prevalent in India compared to Mauritius and the UK due to poorer diabetic control. Diabetes Research and Clinical Practice. 2009 November;86 (2):e39-e40.

94. Seyum B, Mebrahtu G, Usman A, Mufunda J, Tewolde B, Haile S, et al. Profile of patients with diabetes in Eritrea: Results of first phase registry analyses. Acta Diabetologica. 2010 ;47 (1):23-7.

95. Bartels MC, Macheka BM, Guramantunhu S, Scheenloop JJ, Stilma JS. Background diabetic retinopathy in Harare, Zimbabwe. Trop Doct. 1999 Jul;29(3):189-90.

96. Kadiki OA, Roaed RB. Epidemiological and clinical patterns of diabetes mellitus in Benghazi, Libyan Arab Jamahiriya. Eastern Med Health J 1999; 5: 6–13

97. Agaba EI, Agaba PA. Prevalence of microalbuminuria in newly diagnosed type 2 diabetic patients in Jos Nigeria. Afr J Med Med Sci. 2004; 33(1): 19-22.

98. Orluwene CG, Mommoh MO. Screening for microalbuminuria in newly diagnosed type 2 diabetics at a staff clinic in Port Harcourt. Port Harcourt Medical Journal. 2008; 3: 10-14.

99. United nations scheme of geographical region and composition. (Accessed 28th March 2011) http://unstats.un.org/unsd/methods/m49/m49regin.htm

100. Siegfried N, Clarke M, Volmink J: Randomised controlled trials in Africa of HIV and AIDS: descriptive study and spatial distribution. BMJ 2005, 331 [7519]: 742-746.

101. Diabetes Drafting Group. Prevalence of small vessel and large vessel disease in diabetic patients from 14 centres. The WHO Multinational Study of Vascular Disease in Diabetes. Diabetologia. 1985; 28: 615-640.
